# Multimorbidity in Chronic Overlapping Pain Conditions: From Burden to Integrated Care

**DOI:** 10.3390/jcm15124835

**Published:** 2026-06-22

**Authors:** Emmanuel d’Incau, Chelsea Marie Kaplan, Jean-Arthur Micoulaud-Franchi, Christin Veasley, Richard Ohrbach

**Affiliations:** 1 SANPSY, CNRS, UMR 6033, CHU de Bordeaux, Place Amélie Raba Léon, University of Bordeaux, F-33000 Bordeaux, France; jarthur.micoulaud@gmail.com; 2 Chronic Pain and Fatigue Research Center, Department of Anesthesiology, University of Michigan Medical School, Ann Arbor, MI 48109, USA; chelsmar@med.umich.edu; 3 Chronic Pain Research Alliance, Milwaukee, WI 53226, USA; cveasley@cpralliance.org; 4 Department of Oral Diagnostic Sciences, School of Dental Medicine, University at Buffalo, Buffalo, NY 14214, USA; ohrbach@buffalo.edu

**Keywords:** chronic pain, multimorbidity, comorbidity, nociplastic pain

## Abstract

Chronic overlapping pain conditions (COPCs) refer to a set of chronic pain disorders that frequently co-occur and may involve partially overlapping mechanisms. The U.S. National Institutes of Health currently recognizes ten COPCs: fibromyalgia, painful temporomandibular disorders, chronic low back pain, chronic migraine headache, chronic tension-type headache, irritable bowel syndrome, endometriosis, interstitial cystitis/bladder pain syndrome, vulvodynia, and myalgic encephalomyelitis/chronic fatigue syndrome. When multiple COPCs coexist, they are associated with a disproportionate multimorbidity burden, including greater pain, poorer psychological well-being, functional limitations, disability, fatigue, sleep disturbances, diminished quality of life, and increased healthcare utilization. Despite their impact, COPCs remain under-recognized, underdiagnosed, and undertreated. Combining structured literature searches and citation tracking with narrative syntheses, this review examines comorbid relationships, the burden of multimorbidity, and potentially overlapping nociplastic mechanisms. By adopting a multimorbidity-based perspective rather than a one-disease, one-treatment approach, it highlights barriers to care—including limited clinical awareness, under-recognition of additional COPCs, limited mechanistic understanding, and fragmented care—and proposes integrated strategies emphasizing prevention, systematic screening, mechanism-informed assessment, and coordinated, patient-centered multimodal management.

## 1. Introduction

Chronic pain affects nearly one-third of adults worldwide and is a major cause of disability, imposing substantial personal and societal burdens [1]. Many pain disorders co-occur and may involve overlapping mechanisms, giving rise to the concept of chronic overlapping pain conditions (COPCs) [2]. The U.S. National Institutes of Health (NIH) currently recognizes ten COPCs: fibromyalgia (FM), painful temporomandibular disorders (TMDs), chronic low back pain (cLBP), chronic migraine headache (cMHA), chronic tension-type headache (cTTH), irritable bowel syndrome (IBS), endometriosis (ENDO), interstitial cystitis/bladder pain syndrome (IC/BPS), vulvodynia (VVD), and myalgic encephalomyelitis/chronic fatigue syndrome (ME/CFS) [3].

**Figure 1:**
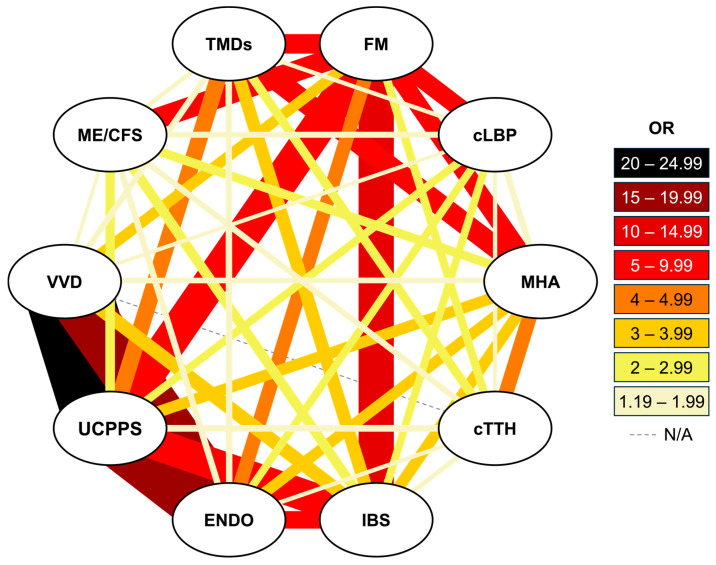
Network of COPCs derived from the Data Direct database (*n* = 687,589), illustrating their comorbid relationships and contribution to multimorbidity—defined as the co-occurrence of at least two chronic conditions in the same individual (adapted from the 2020 analysis reported in [3], with permission from Elsevier). Edge thickness corresponds to odds ratio (OR) magnitude, and edge color indicates OR intervals. Abbreviations: TMDs: temporomandibular disorders; FM: fibromyalgia; cLBP: chronic low back pain; MHA: migraine headache (terminology used in the original source); cTTH: chronic tension-type headache; IBS: irritable bowel syndrome; ENDO: endometriosis; UCPPS: urologic chronic pelvic pain syndrome (i.e., interstitial cystitis/bladder pain syndrome and chronic prostatitis/chronic pelvic pain syndrome); VVD: vulvodynia; ME/CFS: myalgic encephalomyelitis/chronic fatigue syndrome; N/A: OR estimate not available in the original source.

Co-occurrence refers to the presence of two or more disorders without necessarily implying an association, whereas comorbidity refers here to disorders occurring together more frequently than expected from their individual prevalences. COPCs, although defined as separate and distinct disorders, are frequently comorbid (Figure 1), contributing substantially to multimorbidity, which is associated with disproportionately poorer health outcomes, reduced quality of life, and increased socioeconomic costs [4].

Despite their prevalence and impact, COPCs remain under-recognized, underdiagnosed, and undertreated. Limited clinical awareness, insufficient recognition of both comorbidity and the broader multimorbidity profile, limited mechanistic understanding, and fragmented care contribute to suboptimal outcomes, while isolated treatments typically yield only modest benefits [5].

In this review, we describe the clinical features of COPCs, explore their comorbid relationships and multimorbidity burden, and discuss underlying nociplastic mechanisms as well as integrated, patient-centered care strategies aimed at improving prevention, diagnosis, and management.

## 2. Methodology

This review combined two structured and traceable evidence-identification components with several narrative synthesis components. The structured components were limited to Section 3 and addressed (1) pairwise comorbid relationships among the ten COPCs and (2) the clinical impact and overall burden of COPC multimorbidity. The concise clinical overviews of the individual COPCs in Section 3 and the mechanistic, clinical management, and future-oriented discussions in Section 4, Section 5 and Section 6 were developed as narrative syntheses. These narrative components were not based on a formal systematic search or study-selection process but drew primarily on authoritative reviews, consensus statements, clinical guidelines, established diagnostic frameworks, and selected clinically relevant primary studies.

First, a systematic and traceable search strategy, informed by an umbrella review approach, was implemented to identify pairwise comorbid relationships among the ten COPCs. Searches were conducted in PubMed and Scopus from 1 January 1995 to 22 April 2026. Priority was given to English-language reviews of studies conducted in adult populations. When multiple publications addressed the same association, the most recent systematic review was retained unless earlier work provided additional relevant data. Full search strategies and the study selection process are provided in Appendix A and Appendix B.

Second, the clinical impact and overall burden of multimorbidity were assessed through a structured and reproducible secondary citation-tracking procedure. The reference lists of relevant systematic and narrative reviews cited in the manuscript were screened using the same temporal limits, and potentially eligible primary studies were assessed according to predefined eligibility criteria. Inclusion was restricted to peer-reviewed, English-language studies conducted in adult populations with clearly defined chronic COPCs and examining the clinical impact and overall burden associated with the co-occurrence of two or more COPCs. Outcomes of interest included pain, psychological well-being, physical functioning, disability, fatigue, sleep, quality of life, and healthcare utilization. Priority was given to observational studies and large-scale clinical studies involving at least 100 participants. Full eligibility criteria are provided in Appendix A.3.

In addition, a concise and consistent clinical overview of each COPC—including its definition, main symptoms, prevalence, and diagnostic criteria—was based on authoritative reviews, clinical guidelines, and established diagnostic frameworks.

For both structured components, screening and data extraction were performed by the first author. Reports that did not adequately define the chronicity of the COPCs under study were excluded during full-text screening. No formal assessment of methodological quality or risk of bias was conducted. Consequently, the evidence summarized in this review should be interpreted as descriptive rather than as a formally graded body of evidence. The absence of such an assessment limits our ability to compare the methodological robustness of the included studies, to weight individual findings according to study quality, and to draw firm conclusions regarding the certainty or strength of the reported associations. It does not preclude a broad clinical description of the individual COPCs and their commonly reported patterns of comorbidity; however, the magnitude, consistency, and generalizability of specific prevalence estimates and associations should be interpreted cautiously. Residual variability in diagnostic criteria, case definitions, assessment methods, study populations, and periods of data collection further contributes to this uncertainty.

The narrative components were intended to provide an interpretive and clinically oriented synthesis rather than a comprehensive or systematically selected account of all available evidence. They drew on the sources described above and were informed by the authors’ expertise in chronic pain, nociplastic pain, multimorbidity, and integrated care.

## 3. Clinical Features and Multimorbidity in COPCs

In this section, we present the findings from the two structured evidence-identification components concerning pairwise comorbid relationships and the clinical burden of COPC multimorbidity, together with concise narrative clinical overviews of the individual COPCs. The clinical burden of multimorbidity was examined across predefined patient-centered domains, including pain, psychological well-being, physical functioning, disability, fatigue, sleep, quality of life, and healthcare utilization. Patterns reported for specific pairs of COPCs are presented as illustrative of broader combinations that may remain underexplored. The included reviews varied in their diagnostic criteria, case definitions, assessment methods, study populations, and periods of data collection. This residual clinical and methodological heterogeneity may influence prevalence estimates and effect sizes and should be considered when comparing and interpreting associations among COPCs. Accordingly, Table 1 provides a descriptive synthesis of the reported findings and their clinical implications rather than a formally graded assessment of methodological quality, risk of bias, or certainty of evidence.

### 3.1. Fibromyalgia (FM)

This condition is characterized by chronic widespread musculoskeletal pain accompanied by fatigue, sleep disturbances, and cognitive and other somatic symptoms. It affects 2–4% of the population, with a marked female predominance, reflected in female-to-male (F/M) ratios ranging from 2:1 to 9:1 depending on the diagnostic criteria used and the populations studied. Currently, diagnosis most often relies on the 2016 American College of Rheumatology criteria (Widespread Pain Index + Symptom Severity Scale) [26].

FM frequently overlaps with other COPCs, including cLBP [6], ME/CFS [7], IBS [6,8,9], painful TMDs [6,10,11], cMHA [6], and cTTH [6]. Among these, chronic primary headaches (cMHA and cTTH) have a particularly severe impact on individuals with FM [27]. The Orofacial Pain: Prospective Evaluation and Risk Assessment (OPPERA) study further showed that FM is commonly comorbid with cLBP and, even more prominently, with painful TMDs [28]. This multimorbidity is associated with greater pain intensity, functional limitations, and overall symptom burden [29]. Additionally, in individuals with FM, comorbid IBS is associated with greater overall physical symptom reporting than FM alone [30], and health status is further impaired when ME/CFS is also present [31].

### 3.2. Painful Temporomandibular Disorders (Painful TMDs)

TMDs comprise more than 30 musculoskeletal conditions affecting the masticatory system. Painful TMDs, including myalgia and arthralgia, may be acute, recurrent, or persistent. The prevalence of painful TMDs is estimated at approximately 2–7% in adults [32]. In the OPPERA prospective cohort, the annual incidence of first-onset painful TMDs was approximately 4% among adults aged 18–44 years; among incident cases, 12% experienced a single episode, 65% had recurrent episodes, and 19% developed persistent pain. Although the incidence of first-onset painful TMDs is broadly similar in women and men (F/M ratio = 1.3:1), women are at greater risk of chronicity and seek care much more frequently than men [33]. Diagnosis is commonly established using the standardized Diagnostic Criteria for Temporomandibular Disorders (DC/TMD), which includes Axis I (physical examination) and Axis II (psychosocial assessment) [32].

Painful TMDs commonly coexist with other COPCs. Coexisting FM [6,10,11], in particular, is associated with greater TMD-related pain [29,34,35], disability and functional limitations [36,37], psychological symptoms such as anxiety and depression, additional somatic symptoms [34,35,36,37,38], and poorer quality of life [34]. Beyond FM, painful TMDs are also comorbid with cLBP [10,12], cMHA [10,13,14,15], cTTH [14,15], IBS [8,10,16], and ME/CFS [17]. A cross-sectional study indicates that the presence of multiple COPCs, particularly FM, cLBP, cMHA, and IBS, is linked to significantly greater pain-related disability in individuals with painful TMDs [39]. These findings align with the OPPERA study, which showed that a higher number of COPCs among patients with painful TMDs is associated with more severe pain, greater pain interference, more missed activity days [29], increased depressive symptoms [40], and greater functional limitations of the masticatory system [41].

### 3.3. Chronic Low Back Pain (cLBP)

Low back pain is defined as pain, muscle tension, or stiffness below the costal margin and above the inferior gluteal folds, with or without radiating leg pain. When it persists for more than three months, it is considered chronic. Most cases are nonspecific, with no identifiable underlying pathology or nociceptive cause, and may be associated with emotional distress, functional impairment, and reduced quality of life. Prevalence ranges from approximately 12% in adolescents to more than 30% in older populations, with higher rates in women. Assessment may follow NIH criteria addressing pain severity, impact, and comorbidities [42].

cLBP is frequently comorbid with FM [6], cMHA [18], and cTTH [18]. Data from Stanford’s Collaborative Health Outcomes Information Registry (CHOIR), a registry-based learning health system, indicate that comorbidity among these conditions is associated with greater pain intensity and poorer health across somatic, psychological, social, and global domains [43]. Other systematic reviews report a strong association between cLBP and painful TMDs [10,12]; their coexistence is associated with more severe pain characteristics [29]. More broadly, cLBP coexists with at least one other COPC in 45% of cases, most often IBS, followed by ME/CFS and FM. Compared with cLBP alone, this multimorbidity is associated with more severe pain and higher levels of anxiety, depression, and fatigue [44].

### 3.4. Chronic Migraine Headache (cMHA)

This primary headache disorder, defined as a headache occurring on ≥15 days/month for >3 months, with migraine features on at least 8 days/month, affects up to 5% of the population, primarily women. Diagnosis is based on the International Classification of Headache Disorders, third edition (ICHD-3) criteria [45].

Common COPCs among individuals with cMHA include cLBP [18], IBS [19], ENDO [20], and painful TMDs [10,13,14,15]. For example, one systematic review reported that 40% of patients with muscle-related TMDs also had cMHA [13]. The OPPERA study identified migraine as a risk factor for subsequent first-onset painful TMDs [46]. Established painful TMDs were associated with an increased risk of episodic migraine progressing to cMHA in the Chronic Migraine Epidemiology and Outcomes (CaMEO) study [47]. Together, these findings suggest potentially bidirectional temporal associations between migraine and painful TMDs, although they do not establish reciprocal causality. Comorbidity between cMHA and painful TMDs is associated with greater duration and severity of muscle pain [48,49], as well as anxiety and depressive symptoms [34]. The burden is further compounded by FM, which is associated with poorer health outcomes and reduced quality of life [34]. Similarly, individuals with migraine and FM [50] or IBS [51] exhibit higher depression and anxiety severity scores than those with migraine alone, while individuals with migraine and ENDO report greater headache-related disability and more frequent migraine episodes than those without ENDO [52]. More broadly, individuals with cMHA and other COPCs experience greater impairment in pain-related physical and psychological functioning and higher healthcare utilization than those with cMHA alone [53].

### 3.5. Chronic Tension-Type Headache (cTTH)

cTTH is defined as headache occurring on ≥15 days/month for >3 months. Symptoms must include at least two of the following: bilateral location, pressing or tightening quality, mild-to-moderate intensity, and no aggravation by routine physical activity. The prevalence of cTTH is estimated at 2–3%, with an F/M ratio of approximately 2:1. Diagnosis is based on the ICHD-3 criteria [54].

cTTH is frequently comorbid with visceral pain conditions, including IBS, ENDO, and IC/BPS [55], as well as FM [50,56,57], cLBP [18], and painful TMDs [13,14,15]. Although cTTH and painful TMDs are often comorbid, the OPPERA study suggests that cTTH is not a strong risk factor for the initial onset of painful TMDs [46]. However, among patients with painful TMDs, comorbid cTTH is associated with more severe depressive symptoms, greater somatic symptom burden, and greater pain-related disability than painful TMDs alone [58]. This pattern is similar to that observed with comorbid cMHA. In contrast, the coexistence of cTTH and FM does not appear to further increase depression, anxiety, or insomnia compared with FM alone [57]. Nonetheless, patients with both cTTH and FM are more likely to experience depression, anxiety, and insomnia than those with cTTH alone [50].

### 3.6. Irritable Bowel Syndrome (IBS)

IBS is characterized by abdominal pain, bloating, and altered bowel habits and is classified into diarrhea-predominant, constipation-predominant, mixed, or unclassified subtypes. Prevalence in the U.S. ranges from 7% to 16% and is higher in women. Diagnosis is based on the Rome IV criteria [59].

IBS commonly coexists with FM [6,8,9], cMHA [19], ENDO [21,22], and painful TMDs [8,10,16]. Notably, the OPPERA study reported that IBS was comorbid with at least one other COPC in 63% of cases [28]. Individuals with IBS are more than three times as likely to develop painful TMDs as those without IBS [60]. Multivariate analyses show that, when IBS and painful TMDs are comorbid, TMD-related pain severity is closely linked to both abdominal pain intensity and depressive symptoms [60]. Cross-sectional data further indicate that the coexistence of IBS and painful TMDs is associated with greater abdominal pain [61]. Similarly, comorbid IBS and FM are associated with more severe pain-related interference [62], higher levels of widespread pain, sleep disturbances, and fatigue [63], depressive symptoms [64], and poorer quality of life [65,66].

### 3.7. Endometriosis (ENDO)

Endometriosis, now recognized as a systemic disease rather than solely a pelvic disorder, presents with variable symptoms, including pelvic pain, dysmenorrhea, dyspareunia, urinary or bowel pain, and infertility. It affects approximately 5–10% of women of reproductive age and is observed in 50–80% of women presenting with pelvic pain. Diagnosis remains challenging because of nonspecific symptoms and the lack of reliable diagnostic tools [67].

ENDO is frequently comorbid with cMHA [20], IBS [21,22], IC/BPS [23], and ME/CFS [24]. One population-based study found a significant association between ENDO and IC/BPS [68]. Additionally, cross-sectional surveys show that pain intensity is higher when painful TMDs coexist with ENDO than with ENDO alone [69]. Another survey found that 25% of women with ENDO reported at least three additional COPCs—most commonly FM, ME/CFS, and painful TMDs—and that women with multiple COPCs experienced a greater overall pain burden [70]. Overall, women with ENDO and comorbid COPCs are more likely to report greater symptom severity and frequency, poorer pain-related quality of life [71], and higher levels of anxiety and depression [72] than those with ENDO alone.

### 3.8. Interstitial Cystitis/Bladder Pain Syndrome (IC/BPS)

This chronic bladder condition is characterized by urinary urgency and frequency accompanied by pelvic or bladder pain, often with discomfort in the urethra or vagina. Its prevalence ranges from 2.7% to 6.5% among women in the U.S., with onset typically occurring between 30 and 40 years of age. Diagnosis is clinical and made by exclusion, with further testing reserved for atypical or refractory cases [73].

IC/BPS often coexists with other COPCs [74,75]. Notably, it has a strong association with ENDO [68] and particularly with VVD, as highlighted in a systematic review [25]. According to the Multidisciplinary Approach to the study of chronic Pelvic Pain (MAPP) study, IC/BPS is frequently comorbid with IBS, FM, and ME/CFS [76]. This multimorbidity is associated with greater symptom severity [77,78,79] and duration [77], higher rates of depression [77,79] and anxiety [77], and poorer quality of life [77,78,79]. Moreover, as the number of comorbid COPCs increases, stress levels and sleep disturbances increase, while sexual functioning worsens [80]. Comorbid ME/CFS is also associated with a poorer prognosis for IC/BPS symptoms [81].

### 3.9. Vulvodynia (VVD)

This condition is defined as vulvar pain lasting >3 months without an identifiable cause. The pain is often described as a burning sensation and may be localized or generalized, spontaneous or provoked, frequently leading to dyspareunia and sexual dysfunction. Prevalence is estimated at 8–10% among women. Diagnosis is made by exclusion and is supported by the clinical history, pelvic examination, and simple screening questions [82].

VVD frequently coexists with other chronic abdominopelvic pain conditions, particularly IC/BPS [25], while large-scale surveys also report overlap with painful TMDs [83], chronic headaches [83], ENDO [83,84], ME/CFS [83,84], IBS [83,84,85], and FM [83,84,85]. A greater number of coexisting COPCs is associated with greater vulvar pain severity [83] and increased feelings of invalidation and isolation [84]. Comorbid COPCs also affect prognosis: a retrospective study reported that women with VVD and at least one additional COPC were 75% more likely to experience persistent vulvar symptoms [86].

### 3.10. Myalgic Encephalomyelitis/Chronic Fatigue Syndrome (ME/CFS)

This complex condition is marked by severe fatigue, post-exertional malaise, non-restorative sleep, cognitive dysfunction, and often musculoskeletal pain. Prevalence is approximately 1%, with an F/M ratio of 1.5:1 [87]. Diagnosis remains clinical, based on updated NIH criteria, as no validated diagnostic biomarkers are currently available [3].

ME/CFS shows substantial clinical overlap with FM [7], and this comorbidity is associated with a higher risk of major depression and psychiatric morbidity [88]. ME/CFS also frequently co-occurs with other COPCs, including ENDO [24], cMHA, cLBP, IBS, and painful TMDs [89]. According to the Multi-site Clinical Assessment of ME/CFS (MCAM) study, such multimorbidity is associated with greater symptom burden and poorer daily functioning, particularly in activities limited by pain [89].

## 4. Role of Nociplastic Pain in COPCs

### 4.1. Definition

Nociplastic pain is a mechanistic descriptor highlighting altered nociception and the potential importance of central pain-processing mechanisms, which may predominate in COPCs but can occur in any chronic pain condition [90,91,92,93]. In 2017, the International Association for the Study of Pain (IASP) introduced nociplastic pain as a third mechanistic descriptor, alongside nociceptive pain—arising from actual or threatened non-neural tissue damage that activates nociceptors—and neuropathic pain—caused by a lesion or disease of the somatosensory nervous system [90]. IASP defines nociplastic pain as “pain that arises from altered nociception despite no clear evidence of actual or threatened tissue damage causing the activation of peripheral nociceptors or evidence for disease or lesion of the somatosensory system causing the pain”.

These mechanistic descriptors should be distinguished from the classification of chronic pain in the 11th Revision of the International Classification of Diseases (ICD-11). Nociceptive, neuropathic, and nociplastic pain describe mechanisms that may coexist within an individual. By contrast, chronic primary pain is a diagnostic category characterized by persistent pain associated with significant emotional distress and/or functional disability that cannot be better accounted for by another diagnosis. Chronic primary pain frequently involves nociplastic mechanisms but is not synonymous with nociplastic pain and may also include nociceptive or neuropathic components. Nociplastic pain may therefore occur independently or as part of a mixed-pain state, with its severity and relative contribution to the overall pain experience varying along a continuum [90,91].

### 4.2. Neurophysiological Processes

Despite its clinical utility, the concept of nociplastic pain has sparked debate regarding its nosological classification and the broader conceptualization of nociception [94]. For example, nociplastic pain may overlap conceptually with “functional pain” [95], while nociception, considered more broadly, may include nociceptive drive arising from a range of sensory inputs [96,97,98]. Nevertheless, central sensitization is widely recognized as an important neurophysiological process that may contribute to nociplastic pain, although its precise role and relative contribution may vary across conditions and individuals. Central sensitization should not be regarded as a single or universal mechanism underlying all COPCs. Peripheral sensitization—characterized by increased responsiveness and lowered activation thresholds of peripheral nociceptors—may also contribute to or sustain nociceptive drive [99,100,101].

Central sensitization involves altered central nervous system (CNS) processing of sensory input, resulting in heightened responsiveness to both nociceptive and non-nociceptive input, including normally innocuous or subthreshold stimuli. Dysfunction in endogenous pain modulation may accompany this phenomenon and may be associated with CNS hyperexcitability, allodynia (pain in response to normally innocuous stimuli), and hyperalgesia (an exaggerated response to painful stimuli) [102]. However, the extent of altered endogenous pain modulation in COPCs remains uncertain [103].

Beyond amplifying pain itself, nociplastic pain and related alterations in central pain processing are also associated with several non-pain manifestations, including multisensory hypersensitivity, fatigue, sleep disturbances, mood disorders, and cognitive impairment [91,92]. These associated features have been reported to precede or predict the onset and severity of nociplastic pain, supporting their potential relevance for early recognition and future preventive or targeted intervention strategies [104].

### 4.3. Risk Factors

**Figure 2:**
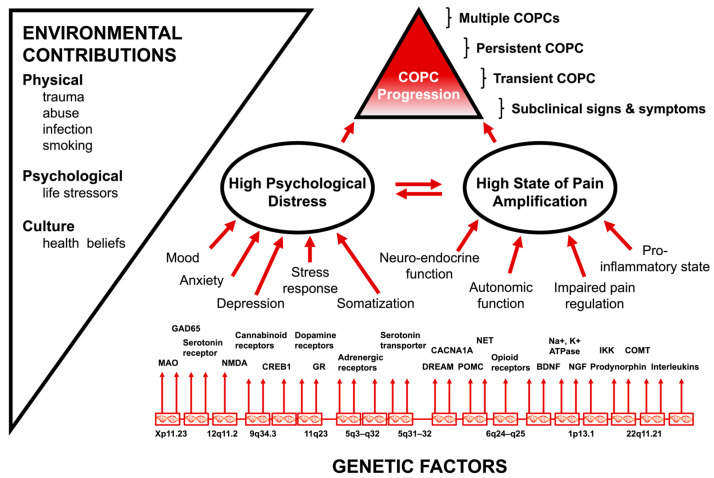
Likely determinants contributing to the risk of onset, maintenance, and progression of common chronic overlapping pain conditions (COPCs). Multiple genetic factors, in combination with environmental exposures, may be associated with increased susceptibility to common COPCs through potential effects on pain sensitivity and psychological vulnerability. Modifiers of the interaction between genetic and environmental factors include sex and ethnicity (adapted from [2], with permission from Elsevier). Abbreviations: MAO: monoamine oxidase; GAD65: glutamate decarboxylase; NMDA: N-methyl-D-aspartic acid; CREB1: cAMP-responsive element-binding protein 1; GR: glucocorticoid receptor; DREAM: downstream regulatory element antagonist modulator; CACNA1A: calcium channel, voltage-dependent, T type, alpha 1I subunit; POMC: proopiomelanocortin; NET: norepinephrine transporter; BDNF: brain-derived neurotrophic factor; NGF: nerve growth factor; IKK: IkB kinase; COMT: catechol-O-methyltransferase.

Susceptibility to nociplastic pain has been associated with a combination of genetic predispositions and environmental influences that may shape individual pain sensitivity and psychological vulnerability (Figure 2) [2,105,106].

The strongest genetic associations involve polymorphisms in genes related to the catecholaminergic pathway, including the Val158Met polymorphism in the catechol-O-methyltransferase (COMT) gene, as well as genes involved in serotonergic signaling, oxidative stress, pain modulation, and inflammation [107]. These genetic variations may be associated with both the number of COPCs and the severity of symptoms commonly attributed to altered central pain processing [108]. Genome-wide association studies have also identified genetic markers that distinguish single-site chronic pain from multisite pain involving COPCs, with stronger genetic contributions observed in the latter [109]. Overall, these genetic influences appear to play a smaller role than environmental and disease-related factors [106].

Environmental influences include early-life experiences, previous pain exposure, lifestyle behaviors, and learned responses [92]. Among these factors, nutritional imbalances—such as a high ratio of pronociceptive omega-6 to antinociceptive omega-3 polyunsaturated fatty acids (PUFAs)—are associated with lower nociceptive thresholds, increased susceptibility to additional COPCs [110], greater pain intensity [111], and elevated somatic and depressive symptoms [112].

While genetic factors alone appear to contribute modestly, their effects may become more pronounced when combined with environmental influences. For example, individuals with COMT-related high pain sensitivity who also report high levels of stress have been found to have a greater risk of developing COPCs [113]. This supports the hypothesis that nociplastic pain may arise from a complex interplay of genetic and environmental determinants, which may contribute to overlapping symptom patterns across COPCs. Although the mechanisms governing susceptibility and resilience remain poorly understood [91,92], findings from OPPERA and related studies suggest that risk factors for nociplastic pain interact rather than act independently [106].

### 4.4. Pathophysiology

**Figure 3:**
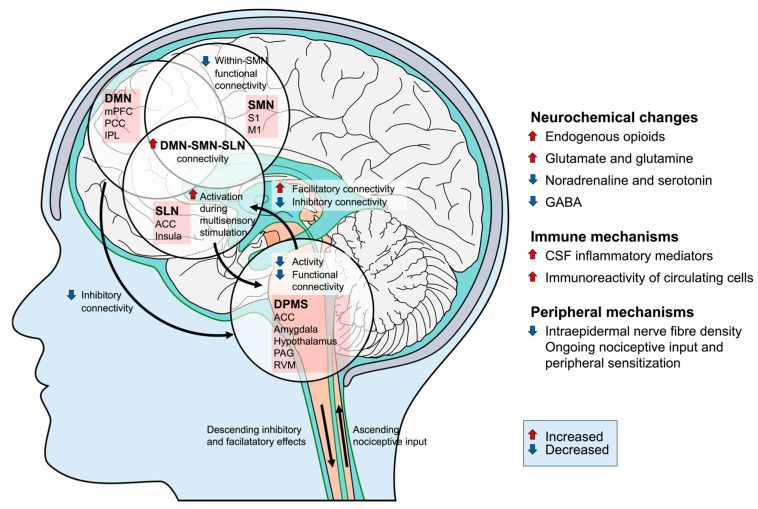
Pathophysiology of nociplastic pain (adapted from [92], with permission from Springer Nature). Neuroimaging studies indicate that nociplastic pain is associated with altered brain connectivity. A widespread distribution of pain has been linked to enhanced functional connectivity among the default mode (DMN), sensorimotor (SMN), and salience (SLN) networks. Altered function of the descending pain modulatory system (DPMS), which regulates nociceptive processing in the spinal dorsal horn through inhibitory and facilitatory pathways, has also been described in nociplastic pain. Alterations involving the immune and peripheral nervous systems have likewise been reported, although their causal significance remains uncertain. Further research is needed to clarify the interactions among these mechanisms in nociplastic pain. Abbreviations: ACC: anterior cingulate cortex; CSF: cerebrospinal fluid; GABA: gamma-aminobutyric acid; IPL: inferior parietal cortex; M1: primary motor cortex; mPFC: medial prefrontal cortex; PAG: periaqueductal gray; PCC: posterior cingulate cortex; RVM: rostral ventromedial medulla; S1: primary somatosensory cortex.

Nociplastic pain has been associated with several potentially interconnected mechanisms, including altered brain connectivity, neuroimmune dysregulation, and peripheral contributions (Figure 3) [91,92,94,99,100,101,102,103,104,114].

#### 4.4.1. Altered Brain Connectivity

Multimodal brain imaging studies have reported altered connectivity patterns involving brain networks associated with pain modulation [92,114]. Specifically, some studies of individuals with COPCs have reported increased functional connectivity involving the default mode network (DMN), sensorimotor network (SMN), and salience network (SLN). In contrast, these networks typically remain distinct or even negatively correlated in pain-free individuals. This enmeshment of brain networks has been associated with widespread pain and greater pain severity, as well as elevated levels of excitatory neurotransmitters, including glutamate and glutamine, and reduced levels of the inhibitory neurotransmitter gamma-aminobutyric acid in key pain-processing regions such as the insula [115]. Paradoxically, enhanced internetwork connectivity may reduce connectivity within the SMN itself [92]. However, these findings vary across conditions and studies; altered network connectivity should therefore be regarded as a potential rather than uniform feature of nociplastic pain [92].

Individuals with nociplastic pain may also exhibit altered function of the descending pain modulatory system (DPMS), which modulates nociceptive transmission in the spinal dorsal horn and, in the orofacial region, in the trigeminal sensory nuclear complex of the brainstem. This system regulates the transmission of nociceptive signals from the periphery to the brain through second-order neurons. Under normal conditions, the DPMS maintains a balance between inhibitory and facilitatory influences. In nociplastic pain, this balance has been proposed to shift toward net disinhibition, which could lower the threshold for nociceptive signaling and contribute to increased pain sensitivity [92]. Key neurotransmitters involved in the DPMS include norepinephrine, serotonin, and endogenous opioids [116]. In cerebrospinal fluid samples from individuals with nociplastic pain, norepinephrine and serotonin concentrations have been reported to be reduced, whereas endogenous opioid levels may be elevated. These reported neurochemical alterations are broadly consistent with clinical observations that drugs enhancing noradrenergic signaling—such as tricyclic antidepressants and serotonin-norepinephrine reuptake inhibitors (SNRIs)—may alleviate symptoms in some individuals with COPCs. In contrast, opioids are generally ineffective and may even worsen symptoms through mechanisms such as opioid-induced hyperalgesia [91,102].

#### 4.4.2. Neuroimmune Dysregulation

Beyond altered brain connectivity, nociplastic pain has been associated with markers of neuroimmune dysregulation, sometimes described as “neuroinflammation,” which may contribute to altered central nociceptive processing [117]. Potential mechanisms underlying these neuronal–glial–immune interactions include elevated levels of inflammatory mediators in cerebrospinal fluid—such as cytokines from the IL-1 family and chemokines—as well as increased activation of CNS glial cells, including microglia and astrocytes [118]. These neuroimmune interactions have been proposed to amplify nociceptive signaling and thereby contribute to heightened pain sensitivity and widespread pain. In parallel, impaired anti-inflammatory pathways may limit the resolution of inflammation, contributing to persistent pain and alterations in mood, motor function, autonomic regulation, and neuroendocrine responses [2,105,106]. However, the causal contribution of glial activation to nociplastic pain in humans remains uncertain [92].

#### 4.4.3. Peripheral Factors

Peripheral factors may also contribute to nociplastic pain [91,92,101,119]. Persistent abnormal input from peripheral nociceptive sources, such as injury or inflammation, as well as altered processing of non-nociceptive sensory input, may generate nociceptive drive [103]. This drive may promote peripheral sensitization and has been proposed to facilitate central sensitization through enhanced CNS pain processing, reduced descending inhibition, and longer-term neuroplastic changes in pain-related networks.

Reduced intraepidermal nerve fiber density (IENFD), which may reflect degeneration or dysfunction of small peripheral nerve fibers (C fibers and Aδ fibers), has been reported in some individuals with nociplastic pain and may be associated with altered peripheral nociceptive signaling [92]. However, reductions in IENFD are nonspecific, and not all individuals with nociplastic pain exhibit these peripheral changes, highlighting phenotypic variability and the variable ways in which these processes may interact over time. Pain in some individuals may be driven primarily by central mechanisms, whereas in others it may involve varying combinations of peripheral and central contributions. Depending on the dominant mechanism, nociplastic pain has been proposed to comprise two putative subtypes—“bottom-up” and “top-down”—which may differentially influence treatment responses [91,92,102].

### 4.5. Sex- and Gender-Related Differences

COPCs and other nociplastic pain conditions are more common in females, particularly after puberty, suggesting a contribution of sex-related biological factors. Proposed mechanisms include differences in pain sensitivity and modulation, hormonal influences, and sex-related patterns of brain network engagement. In nociplastic pain, females may show greater involvement of limbic and affective pain-processing networks, whereas males may exhibit relatively greater engagement of sensory-discriminative networks. However, these patterns remain preliminary, vary substantially across individuals, and are unlikely to explain the female predominance of COPCs in isolation [92,120].

Gender-related factors may also influence symptom reporting, coping, help-seeking, diagnostic recognition, and access to appropriate care. Women’s pain may be more likely to be dismissed, psychologized, or normalized, potentially contributing to diagnostic delays and undertreatment, whereas pain may be underreported or under-recognized in men because of gender norms favoring stoicism and delayed healthcare-seeking. Sex- and gender-related differences in COPCs should therefore be interpreted within an integrated biopsychosocial framework rather than being attributed to a single mechanism [120].

## 5. Integrated Care Strategies for COPCs

Across the studies identified, COPC multimorbidity was associated with poorer outcomes across multiple domains, including pain characteristics [29,30,34,35,39,43,44,48,49,52,53,58,60,61,62,69,70,71,77,78,79,83,86,89], psychological well-being [34,35,36,37,38,40,43,44,50,51,53,58,60,64,72,77,79,80,88], functional capacity [29,36,37,41,43,53,89], disability [36,37,39,52,58], fatigue [43,44,53,63], sleep quality [43,63,78], overall quality of life [34,65,66,71,77,78,79], and healthcare utilization [53].

Beyond the cumulative burden of multiple chronic pain conditions, multimorbidity provides a conceptual framework for rethinking clinical practice, healthcare organization, and research priorities, while raising important ethical challenges [121].

**Figure 4:**
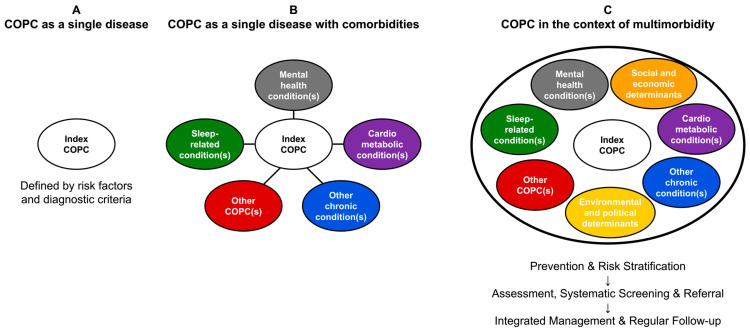
Conceptual and clinical framework for the prevention, assessment, and management of COPCs. Traditionally, the prevention, diagnosis, and treatment of each chronic overlapping pain condition (COPC) have followed a one-disease, one-treatment paradigm (**A**). Although comorbidities are common in patients with an index COPC, management often continues to follow this paradigm (**B**). Based on current evidence, we propose a shift toward a multimorbidity paradigm (**C**), in which patients are viewed as having a unique and dynamic multimorbid profile. Various combinations and degrees of overlap among conditions may occur and fluctuate over time, reflecting the individualized and evolving nature of multimorbidity and its associated burden. Within this framework, clinical care encompasses prevention and risk stratification, initial assessment and systematic screening for additional COPCs and associated symptoms, consideration of predominant pain mechanisms, appropriate referral, integrated multimodal management, and regular follow-up and reassessment. Management should adopt an integrated, transdisciplinary, syndemic-informed approach that considers interactions among conditions and the determinants that shape them, recognizes that no single condition is inherently central, and focuses on what matters most to patients and their caregivers. Care should remain coordinated, minimally disruptive, and aligned with patient values.

To improve the clinical applicability of this perspective, we propose a pragmatic stepwise framework organized around three complementary stages: (1) prevention and risk stratification; (2) assessment, systematic screening, and referral; and (3) integrated management with regular follow-up (Figure 4). This framework is intended as an evidence-informed clinical guide rather than as a formally validated algorithm.

### 5.1. Prevention and Risk Stratification

Primary prevention aims to reduce new cases by addressing causes and risk factors before symptoms appear. For painful TMDs, the U.S. National Academies of Sciences, Engineering, and Medicine (NASEM) recommend screening and risk stratification in primary care and dental settings [122]. The TMD Risk Assessment Tool, developed using data from the OPPERA study [123], categorizes patients as having a low, moderate, or high risk of developing TMD pain over prediction horizons ranging from 6 months to 3 years, based on four domains: psychological symptoms, the presence of other pain disorders (e.g., cLBP and IBS), sleep disturbances, and local orofacial characteristics. At 6 months, the final model showed an area under the curve of 0.75, with 77% sensitivity and 60% specificity; the overall C-index was 0.68. High-risk individuals may benefit from procedural modifications and self-care guidance, although external validation of the tool remains necessary.

For other COPCs, no equivalent tools currently exist. However, certain risk factors for nociplastic pain can be identified [92]. For example, data from a large cohort (UK Biobank, approximately 500,000 adults) indicate that sleep, mood, and cognitive disturbances predict nociplastic pain [124]. In addition, the Risk of Pain Spreading (ROPS) questionnaire evaluates risk factors such as sleep disturbances, neuroticism, mood disorders, life stressors, and body mass index to identify individuals at risk of developing persistent or spreading chronic, high-impact pain, including pain associated with COPCs [125].

### 5.2. Assessment, Screening, and Referral

Primary care physicians are often the first point of contact for patients with chronic pain outside the orofacial region, while dentists commonly encounter patients with orofacial pain and painful TMDs. Early recognition of nociplastic pain may help prevent cascades of unnecessary tests, ineffective treatments, and repeated referrals. In the context of multimorbidity, management requires a structured approach: (1) recognizing chronic primary pain as a distinct diagnostic category; (2) applying a proposed consensus-based diagnostic framework; and (3) systematically screening for additional COPCs.

#### 5.2.1. Recognizing Chronic Primary Pain as a Distinct Diagnostic Category

Traditional classifications, such as the Diagnostic and Statistical Manual of Mental Disorders (DSM) and earlier versions of the ICD, did not adequately incorporate advances in pain neuroscience and offered limited guidance for treatment [126]. A major step forward came with the dual-axis system for TMDs, introduced in 1992 [127], which was later extended more broadly to chronic pain. These developments culminated in the publication of ICD-11 in 2019, which, with guidance from the IASP, formally recognized chronic primary pain as a diagnostic category and a health condition in its own right [126]. This classification should be distinguished from the mechanistic descriptors of nociceptive, neuropathic, and nociplastic pain. Although many COPCs are classified as chronic primary pain conditions and frequently involve nociplastic mechanisms, these concepts are not interchangeable. This paradigm shift helped move chronic pain classification beyond the outdated “biological versus psychogenic” dichotomy, align it with the biopsychosocial model, and reduce stigmatization, particularly among women with COPCs [128].

#### 5.2.2. Applying a Proposed Consensus-Based Diagnostic Framework

In 2021, an IASP Terminology Task Force proposed clinical criteria and a grading system for chronic nociplastic pain, currently limited to musculoskeletal pain [129]. The grading system distinguishes possible from probable nociplastic pain.

Possible nociplastic pain requires four criteria:

1. Pain duration longer than 3 months.
2. Regional or widespread distribution, which may be assessed using body maps.
3. Pain that is not fully explained by nociceptive or neuropathic mechanisms, based on pain descriptors, neurological examination, or specific assessment tools.
4. Evoked hypersensitivity to touch, pressure, movement, or temperature, often disproportionate to the apparent peripheral pathology. Allodynia may be assessed using brushing, palpation, or thermal testing.

Probable nociplastic pain additionally requires:

5. A documented history of hypersensitivity.
6. Relevant comorbidities. These may include hypersensitivity to light, sound, or odors, as well as less specific symptoms such as sleep disturbance, fatigue, and cognitive dysfunction.

This framework represents an essential step toward mechanism-based diagnosis and more individualized treatment strategies.

#### 5.2.3. Systematically Screening for Additional COPCs

Given the cumulative adverse effects of multimorbidity on health and prognosis, systematic screening for additional COPCs is warranted once one COPC has been identified. However, specialty-focused care often overlooks the need for such screening, with providers addressing only the presenting complaint. Consequently, additional pain conditions may go unrecognized, leading to underestimation of symptom burden and suboptimal management [5].

To address this gap, patient advocates supported the development of the Chronic Overlapping Pain Condition-Screener (COPC-S), an electronic tool for clinical and research use that screens for multiple COPCs within 5–15 min using targeted questions and body maps [130]. In a preliminary validation study, the COPC-S showed good overall agreement with physician-administered diagnostic assessments (Cohen’s κ = 0.813; 95% CI, 0.749–0.880). However, it is a screening rather than a diagnostic tool, and further validation in larger and more diverse populations is required.

Referral for condition-specific specialist assessment or coordinated multidisciplinary care should be considered when red flags are present, the diagnosis remains uncertain, symptoms are severe or progressive, substantial functional or psychosocial impairment is identified, or initial management fails to produce meaningful improvement.

### 5.3. Integrated Management and Follow-Up

Management of COPCs aims to reduce pain and its impact rather than necessarily eliminate symptoms, with a focus on functional capacity and quality of life. Setting realistic expectations is essential. Many patients can be managed in primary care, provided that all relevant COPCs are recognized and addressed in a coordinated manner, with referral to specialists for severe or refractory cases. However, few health systems currently provide fully integrated care across specialties, highlighting the need for coordinated and person-centered care [4,5]. Painful TMDs exemplify both these challenges and potential solutions.

#### 5.3.1. Addressing Persistent Gaps

In a U.S. evaluation of practice patterns among dental practitioners, TMDs were primarily managed using a biomedical approach, while the biopsychosocial model remained underutilized [131]. Misconceptions about the complexity of TMDs and the role of self-management, together with reluctance to assess psychosocial factors, contribute to ineffective or unnecessarily prolonged treatments, delayed referrals, and patient frustration—the so-called “medical merry-go-round” [132]. Additionally, access to specialized care is limited, and insurance coverage is inconsistent, thereby adding financial strain [122].

#### 5.3.2. Enhancing Management

Future priorities include expanding access to care, integrating the biopsychosocial model, and strengthening education, collaboration, and research [133]. Within this multimorbidity framework (Figure 4), management should be patient-centered, transdisciplinary, and multimodal [4,5,6,9,10,11,12,13,16,18,19,21,23,24,25]. It should prioritize education, self-management, and non-pharmacological interventions. Pharmacological treatment should be considered selectively according to the predominant pain mechanisms, comorbidities, and individual risk–benefit profile (Table 2) [1,90,91,92,134].

More specifically, nociceptive input remains an important consideration because it may provide persistent or episodic sensory input to the CNS, thereby contributing to the maintenance or amplification of nociplastic mechanisms [135]. Painful TMDs illustrate the therapeutic value of incorporating peripherally directed exercises, such as stretching to improve mobility, while also highlighting the challenge of balancing treatments targeting peripheral contributors with interventions addressing nociplastic mechanisms [136,137].

Finally, regular follow-up should reassess pain and associated symptoms, functional capacity, sleep, psychological well-being, treatment burden, adherence, adverse effects, emerging COPCs, and progress toward patient-defined goals. Management should be adjusted in response to changes in the patient’s clinical and multimorbidity profile.

## 6. Future Directions

While the 2015 white paper on COPCs highlighted a substantial burden for patients and healthcare systems alike [5], important COPC-specific gaps remain. As identified in this review, four areas require particular attention.

First, evidence on comorbid relationships among COPCs remains heterogeneous because of differences in diagnostic criteria, case definitions, study populations, and assessment methods. Future primary studies should use standardized and clearly reported diagnostic frameworks, while systematic reviews should formally assess methodological quality, risk of bias, and certainty of evidence.

Second, longitudinal research is needed to identify predictors of the transition from a single COPC to multiple COPCs and to characterize the trajectories, combinations, and cumulative burden of COPC multimorbidity over time.

Third, the mechanisms specifically associated with COPC overlap remain incompletely understood. Research should clarify whether nociplastic, nociceptive, neuropathic, and neuroimmune mechanisms, as well as sex-related, hormonal, psychosocial, and healthcare-related factors, distinguish individuals with multiple COPCs from those with a single condition.

Finally, treatment strategies require further evaluation in the context of COPC multimorbidity. Future studies should determine how pharmacological and non-pharmacological interventions perform when multiple COPCs coexist, including their effectiveness, safety, interactions among interventions, optimal sequencing, and cumulative treatment burden. Comparative studies are also needed to determine whether integrated, multimorbidity-oriented care provides greater benefit than managing each condition separately.

## 7. Conclusions

COPCs pose major challenges because of their prevalence, frequent comorbidity, potentially overlapping mechanisms, the limited effectiveness of isolated condition-specific treatments, and their substantial burden on patients and healthcare systems. Their management therefore requires a shift from a one-disease, one-treatment model toward a multimorbidity-oriented approach that recognizes the dynamic interactions among conditions, symptoms, mechanisms, and patient priorities. In practice, healthcare professionals should look beyond the presenting condition, screen for additional COPCs and associated symptoms, consider the relative contribution of different pain mechanisms, and coordinate referrals and multimodal care according to each patient’s multimorbidity profile. Breaking down healthcare silos is essential to promote early recognition and deliver coordinated, patient-centered, transdisciplinary, and minimally burdensome care.

## Figures and Tables

**Table 1:** Summary of major findings from systematic reviews assessing COPCs: comorbid relationships and clinical implications.

Author (Year)	COPCs Studied	Main Findings on Comorbid Relationships	Main Clinical Implications Reported by the Review Authors
Kleykamp (2021) [6]	FM cPH TMDs IBS cLBP	Weighted lifetime prevalence estimates for chronic pain conditions involving head/jaw pain, such as migraine (56%), cTTH (48%), and TMDs (57%), were elevated among patients with FM, as were IBS (44%) and cLBP (39%)	Extent of multimorbidity among FM patients will benefit from linking clinical data with administrative claims data; addressing the presence of these comorbid health conditions in clinical trials of treatments for FM would increase the generalizability and real-world applicability of FM research
Ramírez-Morales (2022) [7]	FM ME/CFS	ME/CFS and FM diagnoses overlapped in 47.3% (95% CL: 45.97–48.63) of the reported cases	A prominent clinical overlap between FM and ME/CFS likely reflects shared pathogenetic mechanisms; reported concordance would likely be higher when using the most recent FM diagnostic criteria
Whitehead (2002) [8]	IBS FM ME/CFS TMDs *	The non-gastrointestinal, nonpsychiatric disorders with the best-documented association with IBS are FM (32.5%, 28% to 65%), ME/CFS (51%, 35% to 92%), and TMDs (64%); IBS occurs in 48% (range, 32% to 77%) of patients with FM	Comorbidity of IBS with other disorders is explained by some models as each disorder is the manifestation of varying combinations of interacting physiological and psychological factors
Erdrich (2020) [9]	FM IBS	The overall prevalence of IBS in people living with FM is 46.2% (13.8% to 95.0%); the OR of FM in subjects with IBS is 1.8	Indications that reductions in gastrointestinal symptoms correlate with improvements in FM suggest patients may benefit from identification of the wider range of gastrointestinal disorders and implementation of clinical strategies to address contributing factors
Kleykamp (2022) [10]	TMDs cLBP cMHA IBS FM	Weighted estimates showed high prevalence of comorbid chronic conditions among patients with TMDs, including cLBP (66%), cMHA (40%), IBS (19%), and FM (14%)	Extensive overlap of comorbid pain conditions among patients with different types of TMDs warrants identification of personalized treatment strategies, including the coordination of care across medical specialties
Yakkaphan (2023) [11]	TMDs FM	Meta-analyses yielded a pooled prevalence rate (95% CL) for TMDs in patients with FM of 76.8% (69.5% to 83.3%); almost a third of individuals (32.7%, 4.5% to 71.0%) with TMDs had comorbid FM	TMDs and FM frequently coexist, especially for individuals with painful myogenous TMDs; the clinical, pathophysiologic, and therapeutic aspects underlying this association are important for tailoring appropriate treatment strategies
Justribó-Manion (2024) [12]	cLBP TMDs	The first onset of TMDs was more likely in patients with previous cLBP (HR 1.53, 95% CL: 1.28–1.83); patients with cLBP had 3.25 times the odds of having chronic TMDs compared with those without cLBP (OR 3.25, 95% CL: 1.94–5.43)	cLBP is a risk/contributing factor for painful TMDs; the higher the exposure to cLBP, the higher the risk of developing first onset TMDs; patients with TMDs are commonly treated within a narrow and highly biomechanical paradigm ignoring coexisting cLBP and other conditions, which may influence the prognosis of TMDs
Yakkaphan (2022) [13]	TMDs cPH	The prevalence of TMDs in cPH ranged between 5.12% and 35.22%, while the prevalence of cPH in TMDs was 66.7%	Migraine and tension-type headache are the most prevalent headache disorders associated with TMDs and they often occur in their chronic subtype; this association has important clinical, pathophysiological and therapeutic implications
Réus (2022) [14]	cPH TMDs	Associations between painful TMDs and cMHA or cTTH were reported, with ORs ranging from 40.40 (95% CL: 8.67–188.15) to 95.93 (95% CL: 12.53–734.27)	Recognizing the association between painful TMDs and cPH may help dentists and physicians manage both conditions appropriately or refer patients to a specialist
Bizzarri (2024) [15]	TMDs cPH	The overall risk of TMDs was significantly higher in cMHA populations than in control groups (OR = 24.27, 95% CL: 5.84–100.82) and was also higher in cTTH populations (OR = 3.10, 95% CL: 2.14–4.49)	Migraine and TTH potentially increase the risk of painful, myogenous or combined arthrogenous and myogenous TMDs; possible comorbidities should be considered in the assessment of patients with craniofacial pain
Cricri (2025) [16]	TMDs IBS	Subjects exposed to TMDs had a higher prevalence of IBS signs and symptoms than controls (RR = 3.82, 95% CL: 1.94–7.52); subjects exposed to IBS had a higher prevalence of TMDs than controls (RR = 3.01, 95% CL: 2.03–4.47)	Recognizing the high comorbidity between TMD and IBS can enhance diagnostic accuracy, reducing the risk of misdiagnosis and delays in treatment; routine screening for both conditions in patients presenting with primary symptoms can lead to earlier intervention and improved clinical outcomes; these findings underscore the need for a multidisciplinary approach to patient management
Robinson (2016) [17]	TMDs ME/CFS	In studies of patients with ME/CFS, 21–32% reported having TMDs; studies in people with TMDs reported 0–43% having ME/CFS	The findings suggest substantial potential overlap between the two conditions, but more rigorous methods, including standardized clinical assessments rather than self-reported prior diagnoses, are needed
Vivekanantham (2019) [18]	cPH cLBP	ORs estimating the relationship between cPH (cMHA and cTTH) and cLBP ranged from 1.9 (95% CL: 0.8–4.5) to 9.5 (95% CL: 4.9–18.4)	Patients with comorbid cLBP and cPH may represent a neglected group, highlighting potentially distinct mechanisms and the possible value of combination therapies that could reduce pill burden and medication overuse
Todor (2023) [19]	IBS Migraine	The pooled OR was 2.09 (95% CL: 1.79–2.43) for migraine or headache among individuals with IBS and 2.51 (95% CL: 1.76–3.58) for IBS among individuals with migraine; cohort studies yielded an overall HR of 1.62 (95% CL: 1.29–2.03)	Experimental designs in which therapeutic methods for these conditions can be exchanged or combined may lead to the discovery of more efficient treatment methods
Lechowicz (2025) [20]	ENDO cMHA	cMHA was reported in 64% of women diagnosed with ENDO	A higher comorbidity between ENDO and migraine than expected based on their individual prevalences was observed; specifically, cMHA appeared to be the migraine subtype most strongly associated with ENDO
Saidi (2020) [21]	ENDO IBS	Women diagnosed with ENDO appear to have a two- to threefold higher likelihood of also meeting the criteria for IBS (OR = 2.39, 95% CL: 1.83–3.11); in women initially diagnosed with IBS, some studies reported a threefold risk of having an ENDO diagnosis	Given the close association of the two conditions, both need to be investigated for management of either; gastroenterologists and gynecologists ought to collaborate and develop effective diagnostic and treatment options for these women to avoid medical mismanagement
Nabi (2022) [22]	ENDO IBS	Patients with ENDO have an approximately threefold increased risk of developing IBS (OR = 2.97, 95% CL: 2.17–4.06); the pooled prevalence of IBS in women with ENDO was 23.4% (95% CL: 9.7–37.2)	Doctors should be mindful that patients with ENDO can also have IBS
Inzoli (2024) [23]	ENDO IC/BPS	The association between ENDO and IC/BPS in women with chronic pelvic pain ranged from 15.5% to 78.3%	The diagnosis of both ENDO and IC/BPS requires specific expertise, so women should be referred to a center with a multidisciplinary approach; due to the consistent burden of IC/BPS in women with ENDO, it should be considered in cases of ENDO pain unresponsive to treatment
Compton (2025) [24]	ENDO ME/CFS	The prevalence of ME/CFS among ENDO patients was 17%, while the prevalence of ENDO in ME/CFS populations was 13%; the association of ME/CFS and ENDO yielded a pooled OR of 2.52 (95% CL: 2.45–2.60) and women with ENDO had 2.79-fold higher odds (95% CL: 2.00–3.89) of developing ME/CFS compared to controls	ENDO and ME/CFS may share pathophysiological mechanisms; this underscores the need for integrated care approaches to address overlapping symptomatology in affected patients
Bosio (2024) [25]	VVD IC/BPS	The reported association between VVD and IC/BPS ranged from 51.4% to 94.1%	The two diseases might have similar pathogenetic mechanisms due to their association with the same chronic pain comorbidities; this knowledge is expected to contribute to a deeper comprehension of the two conditions, further facilitating the development of innovative and effective treatments

Note: Diagnostic criteria and case definitions varied across the included reviews and were not consistently reported or analyzed separately; stratification by diagnostic criteria version was therefore not feasible. Abbreviations: FM: fibromyalgia; cPH: chronic primary headaches; painful TMDs: painful temporomandibular disorders; IBS: irritable bowel syndrome; cLBP: chronic low back pain; ME/CFS: myalgic encephalomyelitis/chronic fatigue syndrome; cMHA: chronic migraine headache; cTTH: chronic tension-type headache; ENDO: endometriosis; IC/BPS: interstitial cystitis/bladder pain syndrome; VVD: vulvodynia; COPCs: chronic overlapping pain conditions; OR: odds ratio; HR: hazard ratio; RR: relative risk; CL: confidence limits. * In Whitehead (2002) [8], the term “TMDs” was used without consistently specifying whether the conditions were pain-related; the corresponding estimate should therefore be interpreted with caution.

**Table 2:** Overview of commonly recommended management options for COPCs.

**Non-pharmacological therapies** Patient education, including pain neuroscience education, can help patients understand nociplastic pain as a manifestation of altered pain processing rather than solely a consequence of tissue damage. Self-help resources are also available, including the Chronic Pain Research Alliance patient guide (https://chronicpainresearch.org/wp-content/uploads/2024/12/COPCs-Patient-Guide-web.pdf; accessed on 10 June 2026) and educational websites such as Pain Guide (https://painguide.com; accessed on 10 June 2026). Pain management strategies centered on goal-setting and self-management. Pain-focused CBT with a therapist or through a web-based program. Lifestyle interventions, including individually tailored physical activity or exercise, pacing when post-exertional malaise is present, medical nutrition therapy, stress reduction, and CBT targeting social and vocational re-engagement. CBT and/or ACT for psychological comorbidities, including depression, anxiety, and post-traumatic stress disorder. Sleep interventions, including sleep hygiene, CBT for chronic insomnia disorder, and management of obstructive sleep apnea. Treatment of substance use disorders, including CBT-based interventions. Physical therapy. Acupuncture and dry needling. Other integrative therapies (e.g., yoga, tai chi, qigong, and mindfulness) and related body-oriented self-care practices, such as Pilates, stretching, and strength training. Current evidence does not demonstrate clear clinical benefit from modalities such as TENS, therapeutic ultrasound, and interferential therapy.
**Pharmacological therapies** Centrally acting medications, including tricyclic antidepressants (e.g., amitriptyline), cyclobenzaprine, SNRIs (e.g., duloxetine and milnacipran), NRIs (e.g., esreboxetine), and gabapentinoids (e.g., pregabalin and gabapentin), may be considered following a careful discussion of potential benefits and risks. Importantly, these agents are not universally approved for all COPCs or in all countries and may be prescribed off-label. Simple analgesics and NSAIDs generally provide limited benefit for persistent nociplastic pain but may be considered for the short-term management of pain flare-ups with a nociceptive component. Opioids, benzodiazepines, antipsychotics, and corticosteroid trigger point injections should generally be avoided.
**Potential future therapies** Neurostimulation techniques, such as rTMS or tDCS. Neurofeedback. Cannabinoids and psychedelics.

Note: This table provides a pragmatic overview of commonly recommended management options. The options were not formally graded, and no systematic assessment of the certainty of evidence using the Grading of Recommendations Assessment, Development and Evaluation (GRADE) approach was performed. Abbreviations: CBT: cognitive behavioral therapy; ACT: acceptance and commitment therapy; TENS: transcutaneous electrical nerve stimulation; SNRIs: serotonin-norepinephrine reuptake inhibitors; NRIs: norepinephrine reuptake inhibitors; NSAIDs: nonsteroidal anti-inflammatory drugs; rTMS: repetitive transcranial magnetic stimulation; tDCS: transcranial direct current stimulation.

**Figure A1:**
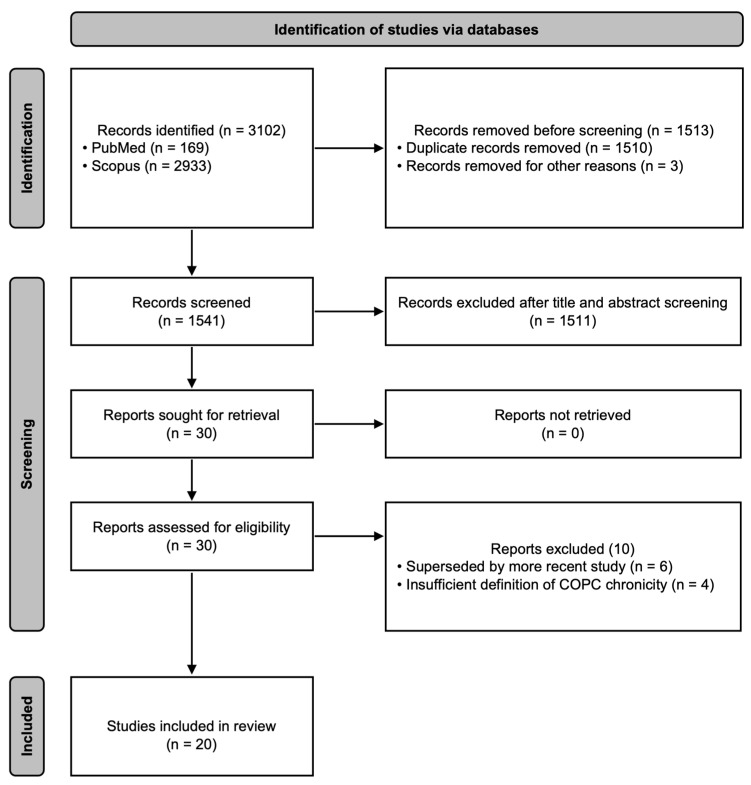
Adapted from the PRISMA 2020 flow diagram.

## Data Availability

No new data were created or analyzed in this study. Data sharing is not applicable to this article.

## Abbreviations

The following main abbreviations are used in this manuscript:

cLBP	chronic low back pain
cMHA	chronic migraine headache
COPCs	chronic overlapping pain conditions
cPH	chronic primary headaches
cTTH	chronic tension-type headache
ENDO	endometriosis
FM	fibromyalgia
IBS	irritable bowel syndrome
IC/BPS	interstitial cystitis/bladder pain syndrome
ME/CFS	myalgic encephalomyelitis/chronic fatigue syndrome
OPPERA	Orofacial Pain: Prospective Evaluation and Risk Assessment
TMDs	temporomandibular disorders
VVD	vulvodynia

## Appendix A. Detailed Literature Search and Identification Strategy for Section 3

### Appendix A.1. Overall Approach

Section 3 comprised two structured evidence-identification components and one narrative clinical overview component: (1) the identification of evidence on pairwise comorbid relationships among COPCs; (2) the identification of studies examining the clinical impact and burden of COPC multimorbidity; and (3) a concise narrative clinical overview of each COPC.

For the first component, a structured and traceable search strategy, informed by an umbrella review approach, was developed to identify systematic reviews and meta-analyses assessing comorbid relationships among COPCs. Although the manuscript was not designed as a formal systematic review, selected elements of the PRISMA reporting framework were applied to improve the transparency and reproducibility of this component.

Systematic searches were conducted in PubMed and Scopus using a tailored Boolean strategy. For each COPC, synonyms and related terms were combined using the “OR” operator, and pairwise combinations between COPCs were constructed using the “AND” operator, resulting in 45 pairwise search queries per database.

To maximize sensitivity and minimize the risk of missing relevant studies, the search strategy was intentionally restricted to COPC-specific terms, without applying additional filters related to comorbidity or epidemiology at the search stage. Language and study-design criteria were applied during screening.

### Appendix A.2. Identification of Study Designs

The identification of systematic reviews and meta-analyses relied on database-specific approaches. In PubMed, validated publication type filters were applied (“systematic review” and “meta-analysis”). In Scopus, relevant studies were identified using a combination of document type filtering and targeted free-text terms (“systematic review” OR “meta-analysis”) in the TITLE-ABS-KEY fields. Study selection and refinement were subsequently performed during the screening stage.

### Appendix A.3. Identification of Studies on the Impact of Multimorbidity

The clinical impact and overall burden of multimorbidity were assessed through a structured and reproducible secondary citation-tracking procedure. The reference lists of relevant systematic and narrative reviews cited in the manuscript were screened, applying the same temporal limits as the database searches. Potentially eligible primary studies were then assessed according to predefined eligibility criteria.

Inclusion was restricted to peer-reviewed, English-language studies conducted in adult populations with clearly defined chronic COPCs and examining the clinical impact and overall burden associated with the co-occurrence of two or more COPCs. Outcomes of interest included pain, psychological well-being, physical functioning, disability, fatigue, sleep, quality of life, and healthcare utilization. Priority was given to observational studies and large-scale clinical studies involving at least 100 participants. Studies that did not report outcomes specifically related to COPC multimorbidity, focused exclusively on pediatric populations, or lacked sufficient information on the study population, COPC definitions, or the outcomes of interest were excluded.

### Appendix A.4. Clinical Overview of Individual COPCs

The concise descriptions of each COPC provided in Section 3—including definition, main symptoms, prevalence, and diagnostic criteria—were based on authoritative reviews, clinical guidelines, and established diagnostic frameworks.

### Appendix A.5. COPCs Included

The following ten COPCs were included:

1. Fibromyalgia (FM)
2. Painful temporomandibular disorders (TMDs)
3. Chronic low back pain (cLBP)
4. Chronic migraine headache (cMHA)
5. Chronic tension-type headache (cTTH)
6. Irritable bowel syndrome (IBS)
7. Endometriosis (ENDO)
8. Interstitial cystitis/bladder pain syndrome (IC/BPS)
9. Vulvodynia (VVD)
10. Myalgic encephalomyelitis/chronic fatigue syndrome (ME/CFS)

### Appendix A.6. PubMed Search Strategy

Searches combined controlled vocabulary (MeSH) and free-text terms searched in the title and abstract fields ([tiab]).

Example structure:

(COPC A terms) AND (COPC B terms)

Full COPC term blocks:

- **Fibromyalgia**

(“Fibromyalgia”[MeSh]

OR “fibromyalgia”[tiab]

OR “chronic widespread pain”[tiab]

OR “diffuse myofascial pain”[tiab]

OR “fibrositis”[tiab])

- **Painful temporomandibular disorders**

(“Temporomandibular Joint Disorders”[MeSh]

OR “temporomandibular disorder”[tiab]

OR “temporomandibular disorders”[tiab]

OR “jaw joint pain”[tiab]

OR “temporomandibular joint disorders”[tiab]

OR “orofacial pain”[tiab]

OR “facial pain”[tiab]

OR “mandibular dysfunction”[tiab]

OR “masticatory system disorder”[tiab]

OR “mandibular disorder”[tiab]

OR “myofascial aching jaw”[tiab])

- **Chronic low back pain**

(“Low Back Pain”[MeSh]

OR “low back pain”[tiab]

OR “lumbago”[tiab]

OR “chronic low back pain”[tiab]

OR “chronic lumbar pain”[tiab]

OR “lumbar pain”[tiab])

- **Chronic migraine headache**

(“Migraine Disorders”[MeSh]

OR “migraine”[tiab]

OR “chronic migraine”[tiab]

OR “chronic migraine headache”[tiab]

OR “status migrainosus”[tiab])

- **Chronic tension-type headache**

(“Headache, Tension-Type”[MeSh]

OR “tension-type headache”[tiab]

OR “chronic tension-type headache”[tiab])

- **Irritable bowel syndrome**

(“Irritable Bowel Syndrome”[MeSh]

OR “irritable bowel syndrome”[tiab]

OR “irritable colon”[tiab]

OR “mucous colitis”[tiab])

- **Endometriosis**

(“Endometriosis”[MeSh]

OR “endometriosis”[tiab]

OR “endometrioma”[tiab])

- **Interstitial cystitis/bladder pain syndrome**

(“Cystitis, Interstitial”[MeSh]

OR “interstitial cystitis”[tiab]

OR “bladder pain syndrome”[tiab]

OR “painful bladder syndrome”[tiab]

OR “interstitial cystitis/bladder pain syndrome”[tiab]

OR “IC/BPS”[tiab])

- **Vulvodynia**

(“Vulvodynia”[MeSh]

OR “vulvodynia”[tiab]

OR “vestibulodynia”[tiab]

OR “vulvar vestibulitis”[tiab]

OR “vulvar pain”[tiab])

- **Myalgic encephalomyelitis/chronic fatigue syndrome**

(“Fatigue Syndrome, Chronic”[MeSh]

OR “chronic fatigue syndrome”[tiab]

OR “myalgic encephalomyelitis”[tiab]

OR “ME/CFS”[tiab])

### Appendix A.7. Scopus Search Strategy

Searches were performed using TITLE-ABS-KEY fields.

Example structure:

(TITLE-ABS-KEY(COPC A terms))

AND (TITLE-ABS-KEY(COPC B terms))

AND (TITLE-ABS-KEY(“systematic review” OR “meta-analysis”))

### Appendix A.8. Screening, Data Extraction, and Reproducibility

Screening and data extraction for the two structured components were conducted by the first author. Because a single reviewer performed these procedures, no formal process for resolving disagreements between reviewers was applicable. Uncertain cases were reassessed against the predefined eligibility criteria.

No formal assessment of methodological quality or risk of bias was performed.

The full list of pairwise search queries, including database-specific syntax, is available upon request. The secondary citation-tracking procedure can be reproduced from the systematic and narrative reviews cited in the manuscript and the eligibility criteria described above.
